# Meteorological Table

**Published:** 1867-04

**Authors:** J. G. Westmoreland

**Affiliations:** Smithsonian Institute, March 1867 at Atlanta, Ga.


					﻿«±» . .....................................................
-o	H fefeW %h» , fee .ee .w ■ • . ,wfc rf
**Q	°* rf rf rf E	OSlrf E E CO E E CO CQ	EEE
S 7"	®
5o 03
--------------------------------—----------------
■to io
<3 •-	...	...
£g^ g a eSS^'^^ .^' • •	. • . .w'~|H^u^h'h’^^h
co -s m	w a k a a z a a	l>
r S rfrfrfrfE EE rf^E	»	rfrfEEEErf
o©
1—< ________________________________________________________
eg ..	----------
.in
*c5	.	......
g	eW-	tHW ,H . . .^0202 . . . .«
50	t— czicdcricci55 55 cdcd #5	»5 J555
l’§)
SCQ ---------------------------------------------------------
.6 0
§'
gW	a"	0101010(=,oio>o©ox5>o®>oo >o>oo®oooo>o>o>o>o>q>oo
M	y—1 rH	r5 tH	t-1 *"5	rl rn rl
•73 5	*
rSA
OQ t-1
fc	_________________________________-
£ o	-----------------
53 E
^2	rf
<2 H	O	«•	O01010oo>ooe©©io>a>oo>oo>o®o®»>ooio>oioo>ci>02
p	«	rlrlrlri	r-1	rlrin	>1
r<^	°	S
A3	O
l^’ £	------------------- ———————
p3
l^-g	a	00101000010000100>000>0®000‘0®C'2'0‘0®'0‘0S
rtriH r^W W rH rH	rH W W	rt
£. '‘-i	-5
2 o	t-
g io
•S °
$0 -y—(	________________________ .	■	■—   —
ts -----------------------------	-;	•
•	S	g	|S§-S ,S .s2 '	’2	1	|
*3	«	£	fiOflOO	PR	«
S g --------------------------—--------------------------------
£ <	H	<3--------------------------------—--------------
o * Sg£gg^SSSS^^^£S33SSSSSS^SSS
ig.	———5-----------------------------------—
g p	|	*’	OS o Ci 00 00 00 00 CO oo co 5S OT 00 OO O O» O 00 00 00 CO oooococibji-jco
-O |	H	—7
§ _J	£	t- O o> CO 00 CO CO COCO co CO CO co GO o Cl Ci CO 00 00 Ci io CM CD CO 00 b^b^CO
*g	S	® O cii S ci ci ci C ci ci ci ci ci ci Ci’ oi oi ci ci ci O* O O ci ci ci O ci Ci’ ci
O	th co co cq qi (M o>j 'M - oqcq<M<M<Mo<iQijoieqQao:iaq<M(McocQaq<MQ4coQ4<Moi
»s o 5	——-------------------------—— ---------------------------------------*—-—
Q> co	S
co	.	t-j o a> o> co co co co co oq co oo co co co a> o o co co co cn	Ci co oq Ci
}	1	o’ ai ci ci Oa ci ai ci ci ci ci oi ci ci ci ci 3s’ oi cri ci ci oi o O ci ci Ci Ci Ci Cl Ci
53 50	t-	CO d (M <M oq <M <M <M aq <M (M <M CM (M	01 eq <M <M Cl <M CO CO <M	<M Cl <M
Day of Month. |’-*e””*‘O«‘-<»<»gSS33SSS3SSa§§^«»«8SSS
				

